# Diffusion-weighted MR imaging in prediction of response to neoadjuvant chemotherapy in patients with breast cancer

**DOI:** 10.18632/oncotarget.18999

**Published:** 2017-07-05

**Authors:** Xue-Ying Hu, Ying Li, Guan-Qiao Jin, Shao-Lv Lai, Xiang-Yang Huang, Dan-Ke Su

**Affiliations:** ^1^ Department of Radiology, Guangxi Medical University Affiliated Cancer Hospital, Nanning, Guangxi Zhuang Autonomous Region, China

**Keywords:** MRI, apparent diffusion coefficient, neoadjuvant chemotherapy, breast cancer

## Abstract

This study aims to evaluate the potential of apparent diffusion coefficient (ADC) derived from diffusion-weighted MR imaging for predicting the treatment response to neoadjuvant chemotherapy (NACT) in patients with breast cancer. Magnetic resonance imaging was performed prior to NACT and after two cycles of NACT. The correlation between mean ADC_pre_ values, mean ADC_post_ values, changes in ADC values and changes in tumor diameters after NACT was examined using Spearman rank correlation. A total of 164 breast cancers were enrolled in this study. Mean ADC_pre_ values of responders ([0.85 ± 0.16] × 10^-3^ mm^2^/s) and non-responders ([0.84 ± 0.21] × 10^-3^ mm^2^/s) had no significant difference (*P* = 0.759). While mean ADC_post_ value of responders was significantly higher than that of non-responders ([1.17 ± 0.37] × 10^-3^ mm^2^/s *vs.* [1.01 ± 0.28] × 10^-3^ mm^2^/s; *P* = 0.002). Both mean ADC_post_ values (r = 0.288, *P* = 0.000) and changes in mean ADC values (r = 0.222, *P* = 0.004) were positively correlated to changes in tumor diameter after NACT, except for mean ADC_pre_ values (r = 0.031, *P* = 0.695). Our results indicated that mean ADC_post_ values and changes in ADC values after NACT might be a biological marker for assessing the efficacy of chemotherapy.

## INTRODUCTION

Neoadjuvant chemotherapy (NACT) is well known to routinely used as standard treatment of breast cancer [1, 2], with the clinical aims of reducing the size of tumors, down staging the disease, improving operative rates, and improving overall survival by the prompt treatment of distant metastases [3, 4]. The accurate and reliable evaluation of response to NACT plays an important role in post chemotherapeutic optimal management, avoiding unnecessary therapy and minimizing drug-related side effects. Traditionally, the evaluation of tumor response has been assessed via tumor size measurements such as clinical examination, X-ray mammography, and ultrasound [5]. However, the assessment of treatment response via the above measurements is considered a relative late event since molecular and cellular changes occur prior to alterations in tumor size [6]. Therefore, a reliable assessment that can provide an earlier indication of therapeutic response is of pivotal importance.

Magnetic resonance imaging (MRI) has been widely used in breast imaging and has been reported as a more effective method for assessing disease extent than physical examination and/or other imaging modalities. And dynamic contrast-enhanced (DCE) MRI has been regarded as a relatively effective tool for assessing tumor progression and/or responses to chemotherapy. DCE-MRI depicts the tumor more accurately through providing information about blood flow and vessel permeability. However, DCE-MRI which is a morphological MR imaging has suboptimal potentiality to distinguish the viable tumor tissue from the tissue of scar, necrosis, fibrosis, and reactive inflammation, and may lead to misestimate the residual tumor size [7, 8]. This potentially yields false positive results and contributes to misrecognize a proportion of responder patients. Therefore, assessing tumor response to NACT could not only depend on DCE-MRI.

Diffusion-weighted (DW) MR imaging is a relatively recent MR imaging sequence that exploits the Brownian motion of water molecules. This technique can be used to measure apparent diffusion coefficients (ADCs)—a quantitative measure of the diffusivity of water—provides information related to tumor cellularity and the integrity of cell membranes and is sensitive to intratumoral changes induced by chemotherapy [9-12]. Nowadays, it was reported in several studies that DW-MRI may be used to evaluate treatment response to NACT in breast cancer patients [13-17]. However, the sample sizes are relatively small and the discriminative ability of a metric tends to be overestimated in those published literatures [13-17]. Therefore, the role of DWI on evaluating the efficacy of chemotherapy in breast cancer still needs further verification. This study aims to assess ADC values in DW-MRI combination with DCE-MRI for evaluating treatment response to NACT in breast cancer patients.

## MATERIALS AND METHODS

### Patients

This study was a retrospective one and was approved by our institutional review board. All patients had signed consent form that their data were used in this study. A total of 164 breast cancer participants were consecutively included in this study. Tumor histology of breast cancer diagnosis and details of estrogen and progesterone receptors were confirmed by core needle biopsy before NACT. All these patients had received NACT before surgery and examined by breast DCE-MRI (1.5T) with DWI before the first cycle of NACT (baseline MRI) and after two cycles of NACT (follow-up MRI) between July 1^st^ 2012 and July 30^th^ 2016. Patients with a metastatic disease or a combination with other cancers which were confirmed by chest X-ray or CT, liver ultrasound or CT, as well as bone scan were excluded.

According to histological classification of breast cancer, 164 cases were comprised of 155 invasive ductal carcinomas, 1 invasive micropapillary carcinoma, 3 invasive lobular carcinomas, 3 metaplastic carcinomas, and 2 unknown. Pre-chemotherapy clinical stages included 4 cases of I, 28 cases of IIA, 76 cases of IIB, 32 cases of IIIA, 10 cases of IIIB, and 14 cases of IIIC. The characteristics of patients were presented in Table 1. All breast cancer patients underwent 4-8 cycles of NACT with the different chemotherapeutic regimen (docetaxel 75 mg/m^2^ + epirubicin 100 mg/m^2^ or fluorouracil 500 mg/m^2^ + epirubicin 100 mg/m^2^ on day 1 and repeated every 21 days). Appropriate surgery was performed in all patients, no matter whether they were responders or not. After the completion of surgery, adjuvant chemotherapy, radiation therapy, and/or hormone therapy were applied.

**Table 1:** Characteristics of patients (*n* = 164).

Characteristics	Responders	Non-responders	*P* value
Total	84	80	
Age (years)	47.7	46	0.289
Pre-chemotherapy clinical stage			0.018
I	1	3	
IIA	21	7	
IIB	36	40	
IIIA	12	20	
IIIB	4	6	
IIIC	10	4	
Tumor histology			0.431
Invasive ductal carcinoma	77	78	
Invasive micropapillary carcinoma	1	0	
Invasive lobular carcinoma	2	1	
Metaplastic carcinoma	3	0	
Others	1	1	
Estrogen receptor			0.211
Positive	52	58	
Negative	27	16	
Unknown	5	6	
Progesterone receptor			0.238
Positive	43	51	
Negative	36	24	
Unknown	5	5	

### MR imaging examination

Breast MR imaging was conducted at a 1.5 Tesla (T) MR imaging system (Magnetom Avanto, Siemens Healthcare, Germany) equipped with a dedicated 8 channel phased array breast coil and subjects in the prone position [18]. The scan parameters were as the previous study [18]. The image parameters of transverse T1-weighted FLASH pulse sequence were repetition time/echo time (TR/TE; 8.6/4.7 ms), section thickness (1 mm), intersection gap (0.2 mm), field of view (32 × 32 cm), and image matrix (323 × 448), respectively. A transverse T2-weighted TIRM pulse sequence was conducted with these image parameters: (TR/TE/inversion time [TI] ), 5600/59/180 ms; section thickness, 4 mm; intersection gap, 0.8 mm; field of view, 34 × 34 cm; and image matrix, 314 × 320. DW-MR images were acquired in transverse planes and covered both breasts with these parameters: b values, 0 and 800 s/mm^2^; TR/TE/TI, 5800/86/180 ms; section thickness, 6 mm; intersection gap, 0.2 mm; field of view, 32 × 32 cm; and image matrix, 323 × 448. For multiphase dynamic contrast enhancement, acquisitions were acquired before contrast agent injection and at approximately 15s after the injection of contrast agents of 0.1 mmol Gd-DTPA (Magnevist meglumine, Bayer Health Care Pharmaceuticals, Germany) per kilogram of body weight. Eight to ten phases were ceaselessly collected and acquisition time for each phase was 55s. The injection rate was 2.0 ml/s, followed by a 20 ml saline flush.

### Measurement of ADC values

DW images and contrast enhanced MR imaging results were reviewed by two experienced radiologists (Guan-Qiao Jin and Shao-Lv Lai, 12 and 16 years of experience in breast MRI, respectively) who were blinded to pathologic findings and treatment responses to NACT. Any disagreements were resolved by a third radiologist (Dan-Ke Su, 20 years of experience in breast MRI).

The ADC values were derived based on the following formula: ADC = -ln[S(b1)/IS(b0)], where S (b1) and S (b0) were signal intensities with (b value of 800) and without diffusion sensitizing gradients ((b value of 0), respectively. Using DCE-MR images as reference purpose, regions of interest in tumor lesions were randomly drawn to extract several circles with 5-10mm in diameter corresponding to the tumor lesion. Meanwhile, the areas with cystic, necrosis, liquefactions, or hemorrhage were excluded. The mean and average ADC were calculated for tumor lesions. The values of tumor ADC before the first cycle of NACT (ADC_pre_) and after two cycles of NACT (ADC_post_) were measured. Changes in ADC values (ΔADC = ADC_post_- ADC_pre_) were also calculated.

### MRI assessment

Treatment response was assessed after two cycles of NACT. Based on the DCE-MRI and Response Evaluation Criteria in Solid Tumors (RECIST) guidelines, patients with breast cancer were divided into responders and non-responders [19]. Responders included patients who had a complete response (CR, complete reduction of tumor lesions) and/or partial response (PR, at least a 30% reduction of the longest diameter of tumor lesions) to treatment. Non-responders included patients with stable disease (SD, less than a 30% decrease or a 20% increase of the longest diameters of tumor lesions) and/or progressive disease (PD, at least a 20% increase of the longest diameters of tumor lesions) to therapy.

### Statistical analysis

Results were presented as mean ± standard deviation (*x* ± SD). Age, pre- and post-NACT mean tumor diameters and ADC values, and changes in ADC values were compared between responders and non-responders using the independent-samples *t* test. Spearman rank correlation was carried to explore the correlation between *(a)* mean ADC_pre_ values and changes in tumor diameters after two cycles of NACT, *(b)* mean ADC_post_ values and changes in tumor diameters after two cycles of NACT, and *(c)* changes in ADC values and changes in tumor diameters after two cycles of NACT. SPSS (version 12.0; SPSS Chicago, III) was used to carry out all statistical analyses. A *P* value of less than 0.05 was considered statistically significant.

## RESULTS

### Characteristics of responders and non-responders

After two cycles of NACT, 164 breast cancer patients were divided into 84 cases of responders (51.2%) and 80 cases of non-responders (48.8%) according to the DCE-MRI and RECIST guidelines. There was no significant difference between responders and non-responders in terms of mean age (responders [47.7 *±* 10.2 years] *vs.* non-responders [46.0 *±* 10.1 years]; *P* = 0.0289; Table 1), as well as mean tumor diameters at pretreatment DCE-MR images (responders [5.2 *±* 2.3cm] *vs.* non-responders [4.6 ± 2.2cm]; *P* = 0.105; Table 1). But pre-chemotherapy clinical stage was significantly different between responders and non-responders (I *vs.* IIA *vs.* IIB *vs.* IIA *vs.* IIIB *vs.* IIIC; *P* = 0.018). After two cycles of NACT, mean tumor diameter in responders was significantly smaller than that in non-responders (responders [2.0 *±* 1.8cm] *vs.* non-responders [4.0 *±* 1.7cm], *P* = 0 .000; Table 2).

**Table 2:** Tumor diameters and ADCs (x ± SD).

Characteristics	Responders (*n* = 84)	Non-responders (*n* = 80)	*P* value
Maximal tumor diameter* (cm)			
Pre-treatment	5.2 ± 2.3	4.6 ± 2.2	0.105
Post-treatment	2.0 ± 1.8	4.0 ± 1.7	0.000
ADC (× 10^-3^ mm^2^/s)			
Pre-treatment	0.85 ± 0.16	0.84 ± 0.21	0.759
Post-treatment	1.17 ± 0.37	1.02 ± 0.28	0.002

ADCs, apparent diffusion coefficients; SD, standard deviation.

*Maximal tumor diameters were measured with DCE-MR images.

### ADC values of responders and non-responders

**Figure 1:**
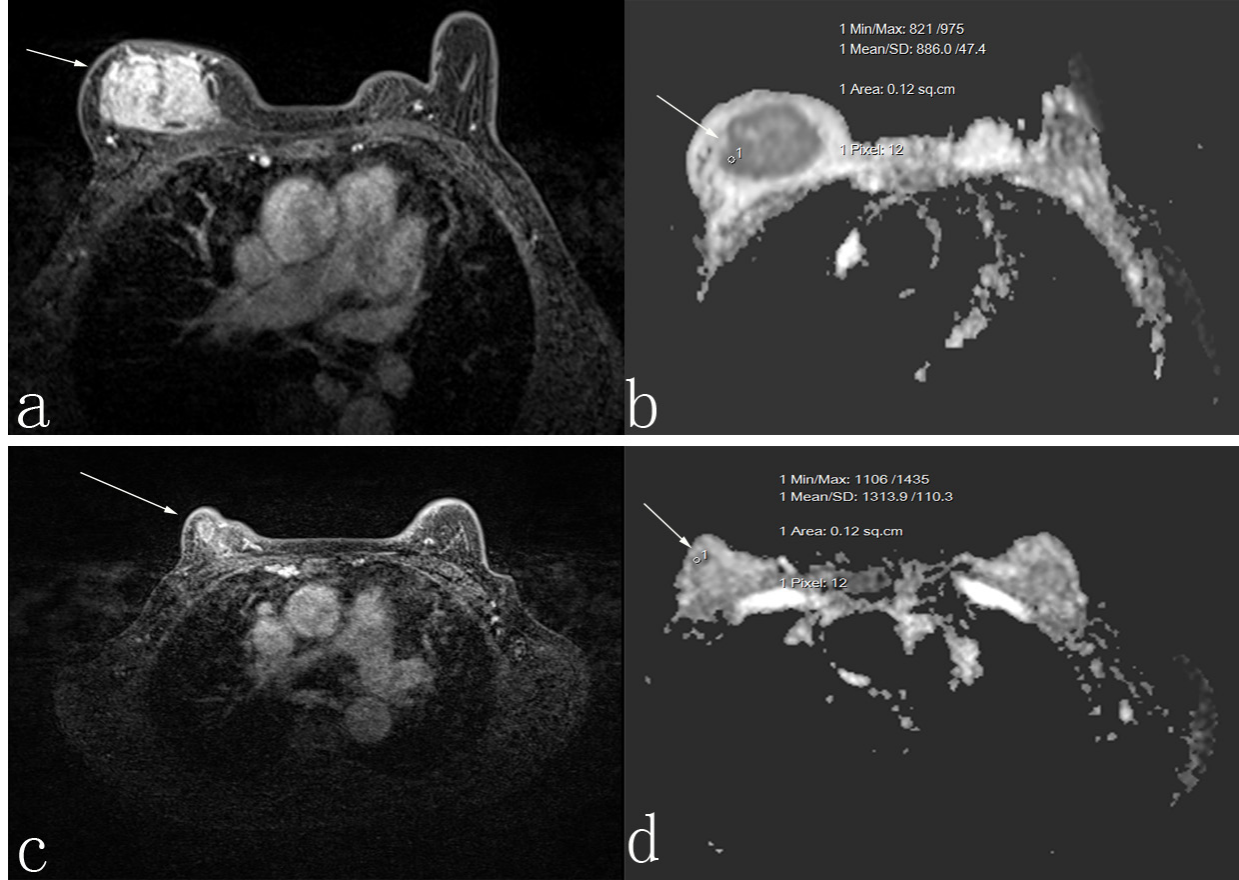
A 50-year-old woman who was responder with invasive ductal carcinoma Before neoadjuvant chemotherapy, the lesion diameter was 5.5 cm in transverse contrast-enhanced T1-weighted image **a.** the apparent diffusion coefficient (ADC) value was 1.118 × 10^-3^ mm^2^/s **b.** After neoadjuvant chemotherapy, the lesion diameter was 3.4 cm in transverse contrast-enhanced T1-weighted image **c.**, and the ADC value was 1.30 × 10^-3^ mm^2^/s **d.**

**Figure 2:**
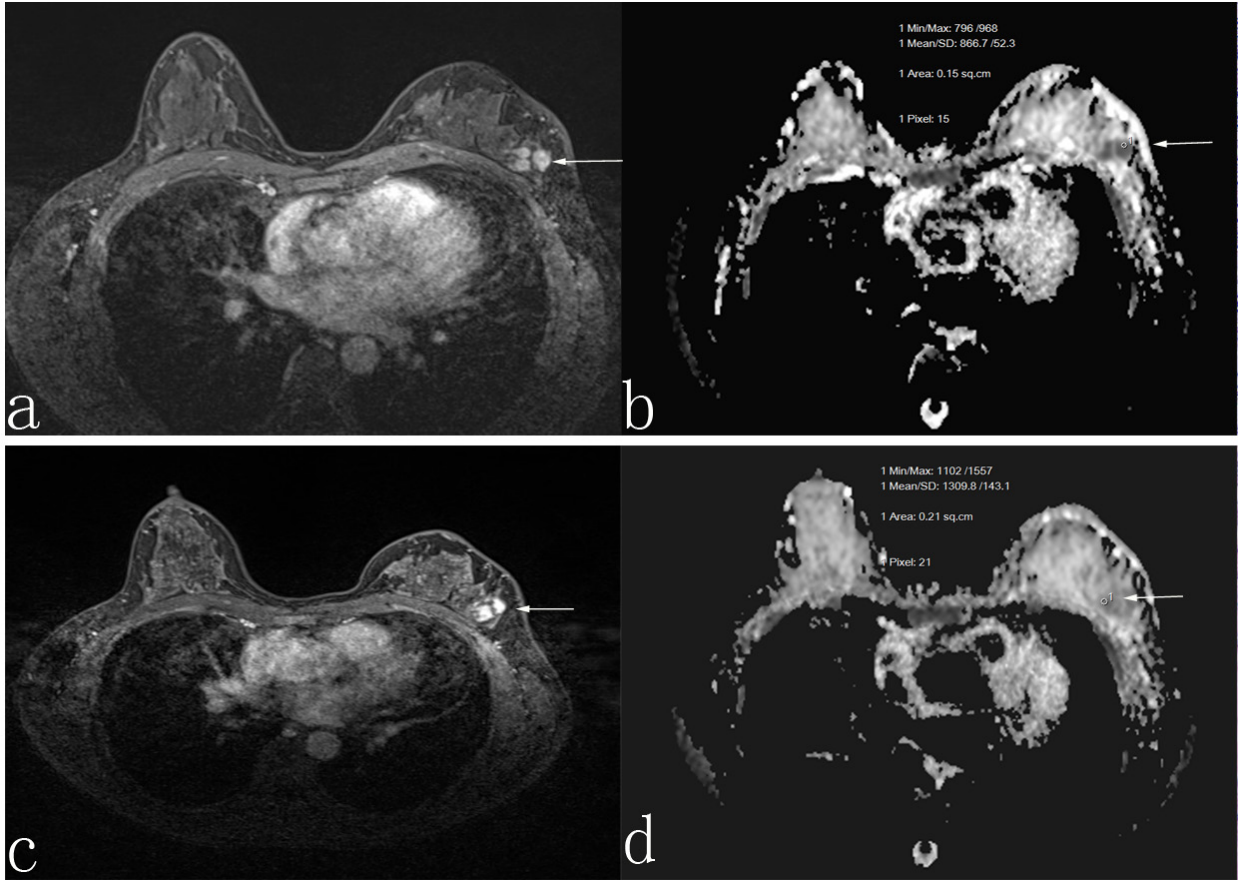
A 45-year-old woman who was non-responder with invasive ductal carcinoma Before neoadjuvant chemotherapy, the lesion diameter was 3.0 cm in transverse contrast-enhanced T1-weighted image **a.** the apparent diffusion coefficient (ADC) value was 1.01 × 10^-3^ mm^2^/s **b.** After neoadjuvant chemotherapy, the lesion diameter was 2.8 cm in transverse contrast-enhanced T1-weighted image **c.**, and the ADC value was 1.06 × 10^-3^ mm^2^/s **d.**

Before NACT, mean ADC_pre_ value of responders ([0.85 ± 0.16] × 10^-3^ mm^2^/s) and non-responders ([0.84 ± 0.21] × 10^-3^ mm^2^/s) had no significant difference (*P* = 0.759). After two cycles of NACT, mean ADC_post_ values significantly increased both in responders (*P* = 0.000) and non-responders (*P* = 0.000) compared with mean ADC_pre_ values. While mean ADC_post_ value of responders was significantly higher than that of non-responders ([1.17 ± 0.37] × 10^-3^ mm^2^/s *vs.* [1.01 ± 0.28] × 10^-3^ mm^2^/s) (*P* = 0.002) (Table 2 and Figure 1-2).

Mean ADC_pre_ values and changes in tumor diameter after NACT were not significantly correlated (r = 0.031, *P* = 0.695), suggesting that there may be no correlation between mean ADC_pre_ values and changes in mean tumor diameter after two cycles of NACT. Mean ADC_post_ values were positively correlated to changes in tumor diameter after two cycles of NACT (r = 0.288, *P* = 0.000), as well as changes in mean ADC values (r = 0.222, *P* = 0.004). The higher the mean ADC_post_ values and the larger the changes in mean ADC values were, the more significant the changes in tumor diameter after two cycles of NACT were.

## DISCUSSION

Pretreatment prediction and early monitoring of treatment response to neoadjuvant chemotherapy is of pivotal importance for developing an optimal management for breast cancer patients. In our study, mean ADC_pre_ values of responders and non-responders had no significant difference. Our results are similar to that of the study briefly reported by Wang et al [20]. While in some previous studies concerning breast cancer, the lower the ADC_pre_ values were, the better the treatment response achieved [13, 14]. Some other studies reported that mean ADC_pre_ values did not predict treatment response to neoadjuvant chemotherapy [21-23]. Classically, the low diffusion values of tumors have been attributed to their increased cellular density [11]. The cellular density of responders may reduce compared with those of non-responders in breast cancer, which may have contributed to the higher ADCs in responders. Hence, breast cancers with higher ADC_pre_ values might be more sensitive to NACT in theory and mean ADC_pre_ value may be a good pretreatment predictor of response to NACT.

In the presented study, with a total of 164 patients undergoing NACT, mean ADC_pre_ values were not correlated to changes in tumor diameter after NACT. Our results are in accordance with that of some previous studies [22, 24]. However, some other published studies found that pretreatment ADC values were negatively correlated to treatment response [13]. The amount of necrosis and necrotic tumors which are hypoxic, acidotic and poorly perfused may explain why tumors are resistance to treatment [25, 26]. Hence, questions concerning the effects of mechanisms underlying breast cancer development and biologic structural differences between breast cancer types on treatment response are worthy of further study.

Our results showed that mean ADC_post_ values significantly increased both in responders and non-responders compared with mean ADC_pre_ values, but mean ADC_post_ values of responders increased more significantly than that of non-responders. Moreover, in our study, mean ADC_post_ values were positively correlated to changes in tumor diameter after two cycles of NACT, as well as changes in mean ADC values. These results are consistent with several previous studies [13-15] which indicated that changes in ADC values after NACT might be associated with tumor response to NACT, as mean ADC values after NACT significantly increase, breast tumor lesions may be sensitive to NACT. The mechanisms of increased ADCs after NACT are described as follows. DW image is well known as a noninvasive examination to reflect biological features of tissue through water diffusion performance shown by ADC values. Water diffusion changes after tissue damage are primarily attributed to changes in volume and curvature of extracellular space which is mainly determined by cell density [27, 28]. Chemotherapy drugs can damage cancer cells directly or indirectly, which result in changes in membrane integrity and permeability of cell membrane rupture, reducing the number of cancer cells and decreasing cancer cell density. Then the extracellular space is expanded, along with significantly increased ADC values.

Several limitations in the present study need to be caution. First, this study was a retrospective study and patients were administrated with different chemotherapeutic regimens, which may have an influence on treatment response. Second, treatment response was based on DCE-MRI measurement of tumor diameter. Although tumor histology after surgery was performed, treatment response after the second cycle of NACT was not confirmed histologically due to different course of NACT. However, both radiologist and surgeons were blinded to pathological report at the time of image analysis. Third, in our study, we set the second cycle of NACT as the time point for early monitoring treatment efficiency, in which most tumor lesions may have morphological changes. Thus, in order to explore the prediction efficiency of ADC values before the morphological change of tumor lesions, we should set an earlier time point in future study, such as 1 day or 1 cycle after NACT. Fourth, breast cancer has four distinct phenotypes and treatment response is closely associated with breast tumor phenotype. Given the small sample, we could not further evaluate the prediction efficiency between different molecular subtype and treatment response. We will further study such an association in the future researches. Fifth, we included six pre-chemotherapy clinical stages (I, IIA, IIB, IIA, IIIB, and IIIC) of breast cancer patients and found that pre-chemotherapy clinical stage was significantly different between responders and non-responders (*P* = 0.018) which might have some influence on our results. Further study with larger-scale was needed to explore such effects.

In conclusion, our results provide evidence that mean ADC_post_ values and changes in ADC values after NACT might be a biological marker for assessing the efficacy of chemotherapy.
